# Predicting cancer stages from tissue energy dissipation

**DOI:** 10.1038/s41598-023-42780-0

**Published:** 2023-09-23

**Authors:** A. Arango-Restrepo, J. M. Rubi

**Affiliations:** 1 Departament de Física de la Matèria Condensada, Universitat de Barcelona, Avinguda Diagonal 647, Barcelona, 08028 Spain; 2 Institut de Nanociencia i Nanotecnologia, Universitat de Barcelona, Carrer Marti i Franques, Barcelona, 08028 Spain

**Keywords:** Bioenergetics, Cancer models, Chemical physics, Statistical physics, thermodynamics and nonlinear dynamics

## Abstract

Understanding cancer staging in order to predict its progression is vital to determine its severity and to plan the most appropriate therapies. This task has attracted interest from different fields of science and engineering. We propose a computational model that predicts the evolution of cancer in terms of the intimate structure of the tissue, considering that this is a self-organised structure that undergoes transformations governed by non-equilibrium thermodynamics laws. Based on experimental data on the dependence of tissue configurations on their elasticity and porosity, we relate the cancerous tissue stages with the energy dissipated, showing quantitatively that tissues in more advanced stages dissipate more energy. The knowledge of this energy allows us to know the probability of observing the tissue in its different stages and the probability of transition from one stage to another. We validate our results with experimental data and statistics from the World Health Organisation. Our quantitative approach provides insights into the evolution of cancer through its different stages, important as a starting point for new and integrative research to defeat cancer.

## Introduction

**Figure 1:**
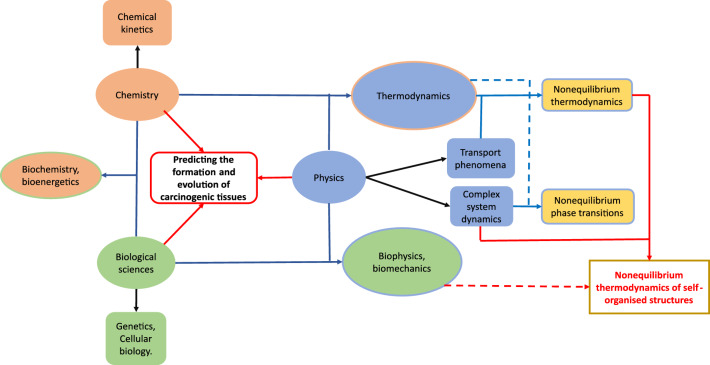
Different scientific areas contributing to the prediction of carcinogenic tissue formation. The union of some fields of these areas might bring new and more robust approaches to understanding the emergence of carcinogenic tissues.

According to the World Health Organisation, about 10 million people suffered from cancer in 2020, the 0.13$\%$ of the world’s population^1^. Statistical projections indicate that this percentage could double by 2030^2^. Although great efforts are currently being made to map in detail the genetic and biochemical alterations that occur in cancer, it is becoming increasingly clear that it is difficult to integrate and interpret the data and translate it into treatments^3^. It is therefore urgent to consider new approaches that provide information on the emergence and sustainability of cancerous tissues in order to apply the most appropriate therapies. Figure 1 shows that understanding the emergence, sustainability and evolution of cancerous tissues is a multidisciplinary issue.

Physical laws are essential for a thorough understanding of the occurrence and progression of cancer at all length scales. Modelling based on these laws provides quantitative aspects useful for understanding a process as complex as cancer development. In particular, one branch of physics, thermodynamics, provides information on the mechanism of cancer emergence and growth, from proteins to cells^4–7^. Analysing the mechanobiology of cancer using a thermodynamic formalism^8,9^, helps to reveal why certain changes in cell and tissue architecture are so useful in detecting, identifying and staging cancer. The concept of entropy production, borrowed from non-equilibrium thermodynamics, has been used to explain the different mechanisms occurring in cancerous tissues at different scales and stages^10–13^. Therefore, thermodynamic models can provide insights into cancer development^4–7,12,14^.

Tissues can be understood as self-assembled (SA) and self-organised (SO) structures that form under non-equilibrium conditions, with consequent entropy production and energy dissipation, and with a response to external stimuli that depends on the intimate properties of the structures^15–19^. It has been suggested that the evolution of cancer through its successive stages and its metastatic tendency could be interpreted as a non-equilibrium phase transition involving energy dissipation^14,20^. Since the study of the emergence of cancer and its evolution through natural selection does not provide conclusive results because of whether it is to the benefit of the cell or the organism^21^, we want to contribute to a better understanding of this important topic by using non-equilibrium thermodynamics as a promising tool.

**Figure 2:**
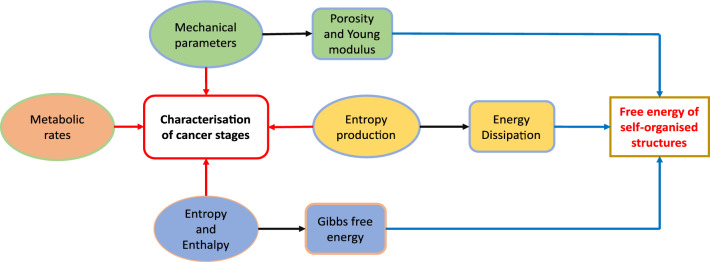
Thermodynamic characterisation of cancer tissues through their mechanical parameters, entropy production and non-equilibrium free energy from which the transition between the different stages of cancer can be analysed.

Cancer tissues are made up of cells with high self-replication rates that can adapt to the environment to survive^22^. Cancer therapies (chemotherapy, radiotherapy, immunotherapy, ozone therapy, hyperthermia and ultrasound therapy) involve the intervention of external agents such as chemical and mechanical forces that dismantle the cellular structure and strip it of cancer cells. The presence of such forces causes the tissue to dissipate energy^23,24^, the amount of which depends on the physical properties of the tissue which in turn provide information about the stage of cancer, as well as the tendency to metastasise^25,26^. Changes in cellular and extra-cellular mechanical properties can promote the growth of cancer^27^, in which the connection between mechanical and biochemical aspects is a powerful tool to develop diagnosis and therapies^28^.

In this article, we show that cancer stages can be predicted from knowledge of the energy dissipated at each stage. To calculate this energy, we first analyse the dynamic response of the tissue to external agents, such as mechanical or chemical forces associated with treatment, taking into account that the tissue is characterised by its porosity and elasticity, and then calculate the entropy production from the non-equilibrium thermodynamics of the tissue. The results obtained allow us to understand why cancerous tissues at different stages adopt certain values of porosity and elasticity, key properties for the development of therapies. We analyse the configurations that dissipate more energy and relate them to the reported data on pancreatic adenocarcinoma. From the characterisation of the different stages of cancer in terms of the energy dissipated, we thus estimate the transition probabilities between them. This perspective allows us to understand the evolution of cancer as a function of a few global tissue parameters. Figure 2 summarises the different steps leading to the thermodynamic characterisation of cancerous tissues. The scheme shows that, by using experimental values for the porosity and Young’s modulus of the tissue, the energy dissipation is obtained which is a measure of the energy needed to change the tissue structure during its evolution. This quantity plays a central role in quantifying the occurrence and sustainability of cancerous tissues.

## Results

We have calculated the energy dissipated by a cancerous tissue when a traction force is applied to it, and from this the probability of a tissue configuration and the probability of its evolution through the different cancer stages (see Methods).

**Figure 3:**
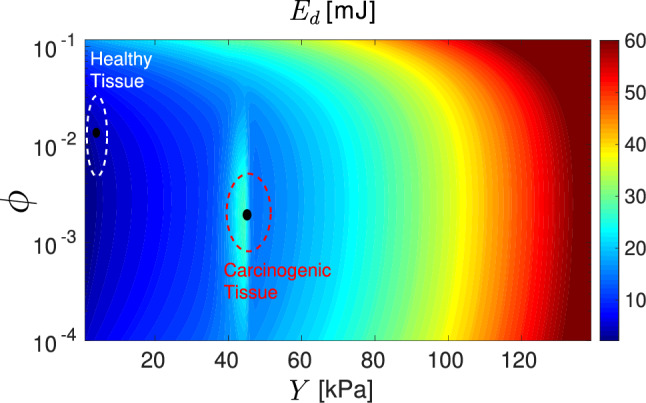
Energy dissipated per gram of tissue $E_{d}$ [mJ] as a function of Young’s modulus *Y* [kPa] and porosity $\phi$ (dimensionless). The values obtained from our model are given by the colour map. The experimental data for healthy tissue and pancreatic adenocarcinoma tissue correspond to the regions delimited by the dashed lines^29,30^.

Figure 3, shows (black dots) the values of porosity and Young’s modulus corresponding to healthy and carcinogenic pancreatic tissues obtained in the experiments^29,30^. The color map of the figure shows that the energy dissipation is greater in pancreatic adenocarcinoma than in the healthy tissue in agreement with the experimental results corresponding to the regions delimited by the dashed lines of the figure. Cancerous tissues then dissipate more energy than healthy tissues. We also see that the energy dissipated is larger at low porosity and high Young’s modulus. These results explain the fact that higher values of energy dissipated in cancerous tissues result in higher rates of self-replication and self-repair processes that take place outside thermodynamic equilibrium. In contrast, healthy tissues show a mild response to external stimuli which is explained by the fact that the cells do not perceive the stimuli as an attack.

**Figure 4:**
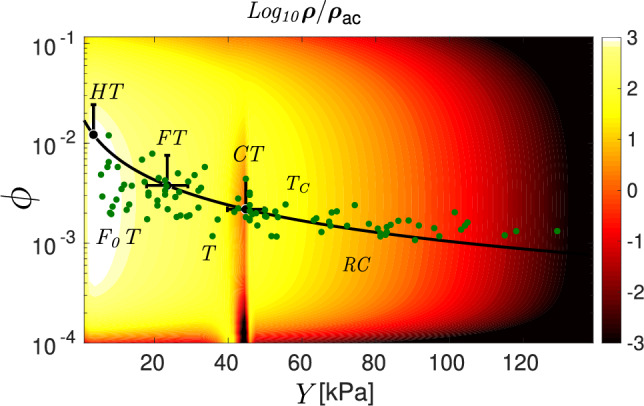
Probability of a tissue configuration, $\rho$, as a function of Young’s modulus *Y* and porosity $\phi$, scaled to the probability $\rho _{ac}$ corresponding to the average value of the parameters of cancerous tissues reported in Ref.^29^: $\phi =0.0022$ and $Y=45.5$ kPa. Values obtained from our model are given by the colour map. Green points correspond to pancreatic adenocarcinoma experimental data^29,30^, whereas the continuous black line shows the trend of these data. The figure shows the different stages: healthy tissue (HT), early fibrous tissue (F$_0$T), fibrous tissue (FT), transition from fibrous to carcinogenic (T), carcinogenic tissue (CT), transition from carcinogenic to rigid (T$_{c}$) and rigid carcinogenic tissue (RC).

In Fig. 4, we represent the probability of a tissue configuration $\rho$ as a function of porosity and Young’s modulus scaled with the probability $\rho _{ac}=\rho (\phi _{c}, Y_{c})$ corresponding to the average value of porosity and Young’s modulus observed in pancreatic adenocarcinoma: $\phi _{c} =0.0022$ and $Y_{c} = 45.5$ kPa. We observe that it is very unlikely to find a tissue with low porosity and high Young’s modulus^29,30^. The experimental values of $\phi$ and *Y* are given by the green dots with the black line indicating their tendency. They correspond to healthy, fibrous, carcinogenic and rigid carcinogenic tissues, as well as an initial fibrous stage located between the healthy and fibrous stages. The probability reaches its highest values as $\phi$ increases and *Y* decreases (healthy tissue). The initial stage of fibrosis, which corresponds to the white region lies in the vicinity of the local maximum of $\rho$. The local minimum of $\rho$ takes place at the average values $\phi =0.0022$ and $Y = 45.5$ kPa, whereas the region for which $\rho$ decreases corresponds to rigid carcinogenic tissue. In the figure, we also see that $\log _{10}{\rho /\rho _{ac}}$ for healthy tissues is 3, i.e., it is 1000 times most likely to be healthy than cancerous.

**Figure 5:**
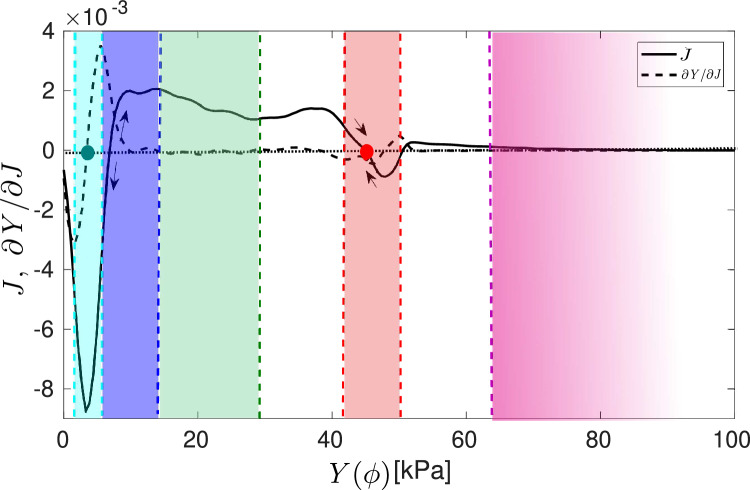
Probability current *J* [1/s] and its derivative $\partial J/\partial Y$ as a function of Young modulus *Y*. Cyan, blue, green, salmon and magenta regions correspond to healthy, initial fibrosis, fibrosis, carcinogenic and advanced carcinogenic stages. White regions represent unlikely configurations for the corresponding values of *Y* in pancreatic adenocarcinoma. Blue and red points denote healthy and carcinogenic tissues.

In Fig. 5, we plot the probability current and its derivative as a function of Young’s modulus. The cyan region corresponds to the healthy stage, while the blue region denotes the initial fibrosis stage. The green region represents the fibrosis stage, while the Young’s modulus values corresponding to the white regions are highly unlikely in pancreatic adenocarcinoma. The light red region is the typical carcinogenic stage of most cancers, while the magenta region represents the advanced carcinogenic stage.

We observe that in the healthy and fibrosis stages it is fulfilled that $\partial J/\partial Y = 0$, so they are dynamically stable states (see Methods). In the fibrosis and transition stages, the current is positive, implying a high probability of transition favouring the cancer stage. Furthermore, in the cancer stage the sign of the current changes from positive to negative, which means that there is a configuration to which the system will tend once the process has started. Finally, the transition from cancer to advanced cancer regimes tends to be slow as the current is very small. The advanced and rigid cancer stage is dynamically stable as the derivative is approximately zero.

**Figure 6:**
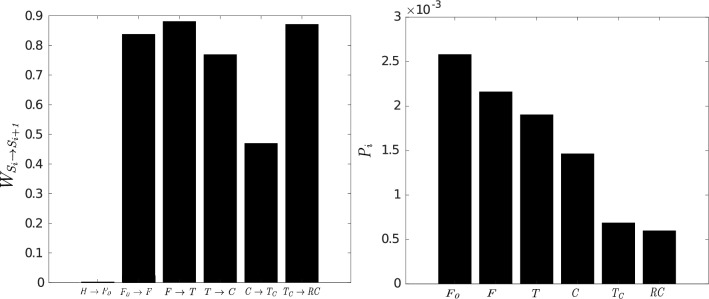
Transition probability between cancer stages and conditional stage probability. The left-hand side figure shows the transition probability from healthy to initial fibrosis ($H\rightarrow F_{0}$), initial fibrosis to fibrosis ($F_{0}\rightarrow F$), fibrosis to transition stage ($F\rightarrow T$), transition stage to cancer ($T\rightarrow C$), cancer to carcinogenic transition stage ($C\rightarrow T_{C}$) and from carcinogenic transition stage to advanced/rigid cancer ($T_{C}\rightarrow AC$). The right-hand side figure shows the conditional stage probability for initial fibrosis $F_{0}$, fibrosis *F*, transition *T*, cancer *C*, carcinogenic transition $T_{C}$ and advanced cancer stage *RC*.

In Fig. 6, we show the transition probability (left side) and the conditional probability (right side), defined in Methods. A very low value for the probability of transition from the healthy stage (H) to the early fibrosis stage ($F_{0}$) is observed, as expected from the analysis of the probability current. The transition probabilities are higher than 0.75 except that from the carcinogenic stage (C). This means that once the tissue has reached the initial fibrosis stage ($F_{0}$), the evolution towards a carcinogenic stage (C) is energetically favoured, whereas that to a more advanced cancer stage, ($T_{c}$ or *RC*), is less favoured. The results obtained for the conditional probability show that, beyond the healthy stage, it decays rapidly becoming lower than 25 in 10000. We have found that the probability of observing pancreatic tissue at the cancer stage is approximately 1/1000, according to World Health Organisation data^1^.

## Discussion

We have proposed a general model that describes the evolution of a cancerous tissue through its different stages, from healthy to carcinogenic tissue, focusing on the case of pancreatic adenocarcinoma. The tissue is considered as a self-organised structure that can be characterised by mechanical parameters such as porosity and elasticity, and by the dissipated energy (computed from Eqs. (10)–(12)) obtained from the dynamic response of the tissue to an external force (by solving Eqs. (1)–(9)). From the energy dissipated, we have computed the non-equilibrium free energy by means of non-equilibrium thermodynamics of the formation of self-assembled structures^31^ (Eq. (15)) and from it the probability of transition between the different stages of the cancerous tissue (Eq. (19)). The proposed predictive model allows us to know both the evolution of cancer and the level of treatment required using Young’s modulus value of the patient’s tissue.

Our study has demonstrated that healthy tissues operate with minimal energy dissipation, resulting in thermodynamically efficient SA/SO structures. In contrast, cancerous tissues function with maximum energy dissipation and possess an effective dynamic response to external stimuli. These scenarios are frequently observed in physical-chemical and biological systems.

Because carcinogenic tissues are highly dissipative structures, it is not a good strategy to use therapies that increase energy dissipation. Future treatments should consider external agents that interact with the carcinogenic tissue and result in low-energy dissipation, such as drugs that do not induce significant changes in free energy.

Our work aligns with previous research, which has considered cancer cells and tissues as out-of-equilibrium systems that generate entropy and dissipate energy due to irreversible processes^32–34^. Our study, along with previous research, has successfully measured the effect of external forces on the thermodynamic and dynamic responses of these systems by calculating entropy production. This collective body of work supports our current proposal in the field of biothermodynamics, which aims to enhance our comprehension of cancer dynamics.

## Conclusions

Our work presents a methodology that utilizes thermodynamic results, specifically energy dissipation, to gain insight into cancerous tissues. Our model accounts for the conservation of matter and energy, as well as important system variables. Further variables and phenomena can be added to improve accuracy and delve deeper into cancer dynamics, including growth, metastasis, and a biochemical analysis of metabolic pathways from a bioenergetic and biothermodynamic standpoint.

The proposed model and methodology allow, in particular, to understand the evolution of pancreatic adenocarcinoma and its different stages without the need to invoke complex evolution equations for Young’s modulus and porosity that characterise the structure. The energy dissipation study carried out could be extended by considering different metabolic pathways. In this way, the role of dissipated energy could be analysed more precisely and thus provide a more detailed description of the evolution of cancerous structures and an assessment of the prospects offered by the proposed treatments.

## Methods

To characterise the different tissue configurations from the energy dissipated, we will first analyse the response of the tissue to a force and obtain the resulting entropy production for different values of porosity and Young’s modulus which multiplied by the temperature gives the energy dissipation. We will subsequently obtain the non-equilibrium free energy of the self-organised structures and from it the probability that the tissue adopts a given configuration corresponding to the different cancer stages.

Specifically, we will study the tissue dynamic response to both a periodic mechanical force $F_{m}$ that modifies the space between cells and a chemical force $F_{ch}$, which affects the flow of chemical compounds such as glucose or drugs through the tissue.

**Figure 7:**
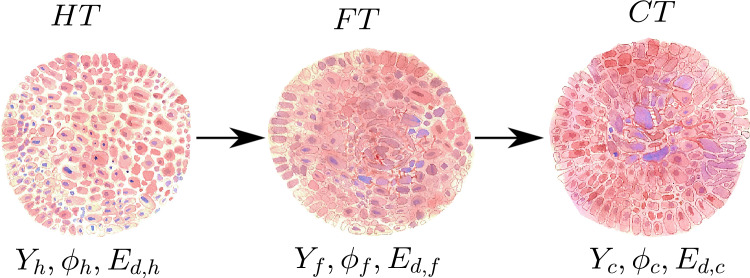
Three possible tissue configurations composed of cells, extracellular matrix, and interstices. Healthy tissue (HT) shows a more ordered distribution of cells and less tortuous cell spacing than cancerous tissue (CT) while fibrous tissue (FT) is an intermediate structure between HT and CT. The tissue configurations are quantified by porosity and Young’s modulus: $\phi _{h}$ and $Y_{h}$ for HT, $\phi _{F}$ and $Y_{F}$ for FT, and $\phi _{c}$ and $Y_{c}$ for CT. In healthy tissues, Young’s modulus is lower and porosity is higher than in carcinogenic tissues. Tissues can also be characterised by the dissipated energy: $E_{d,h}$ for healthy tissues, $E_{d,F}$ for fibrous tissues and $E_{d,c}$ for carcinogenic tissues.

### Tissue structure

Tissue microstructure can be characterised primarily by its porosity $\phi$, which measures the volume fraction of the extracellular space, and its Young’s modulus *Y*, related to the tissue stiffness, proportional to the elastic constant. Although in principle many different configurations are compatible with a given value of these two parameters, only a few of them have been observed. Figure 7 shows three possible configurations: healthy tissue, fibrous tissue and carcinogenic tissue. They have different porosity, Young’s modulus, and dissipated energy $E_{d}$ which we will define in the next subsection.

**Figure 8:**
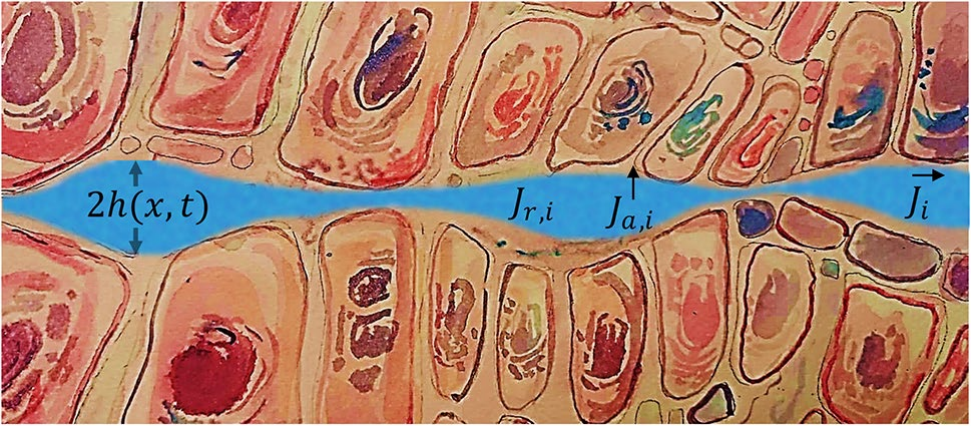
Illustration of an intercellular space of varying half-width *h*(*x*, *t*) [m] through which drugs can flow, with $J_{i}$ [mol/m$^2$s] mass fluxes. Compounds can also be absorbed at rates $J_{a,i}$ [mol/m$^3$s], and consumed at rates $J_{r,i}$ [mol/m$^3$s].

We consider the intercellular space to be formed by channels of variable width, as shown in Fig. 8, due to the periodicity of the applied force which can be modelled as

1 $$\begin{aligned} h(x,t) = A_{h}\left( \sin \left( \pi \frac{x}{R_{m}} - \frac{\pi }{2}\right) +1\right) |\sin (ft)| + h_{0} \end{aligned}$$

where *x* is the position along the channel in m, *t* the time in s, $A_{h}$ the amplitude of the channel in m, $h_{0}$ the bottle neck width in m, $R_{m}$ the characteristic length of the channels in m (4 cells diameter^31^) and *f* the frequency of the applied force in s$^{-1}$. The amplitude is a function of the porosity defined as $\phi = \frac{1}{R_{m}^{3}} \int _{0}^{R_{m}} h^{2} dr$. From this expression, using Eq. (1) and solving for $A_{h}$, we obtain

2 $$\begin{aligned} A_{h} = \sqrt{(2/3)\phi R_{m}^2 - (2/9)r_0^2} - (2/3)r_0 \end{aligned}$$

where $r_{0}$ [m] is the smallest opening in the walls of the capillaries that carry blood and chemicals to the tissues.

To compute $h_{0}$, we consider that the amount of energy needed to expand the channel a distance $h_{0}-r_{0}$ is the elastic energy $E_{e} = Y(h_{0}-r_{0})^{2}/2R_{m}$ [J]^35^. We next assume that the energy given by the mechanical external force $F_{m}$ is proportional to the pressure difference induced inside the capillary $\Delta P$ [kPa]: $E_{F}(F_{m}) = C_{0} \Delta P$. By equating $E_{e}$ with $E_{F}$, we thus find

3 $$\begin{aligned} h_{0} = \sqrt{\frac{2C_{0}R_{m} \Delta P}{Y}} + r_{0} \end{aligned}$$

where the constant $C_{0}=5\times 10^{-10}$m$^{3}$ can be estimated from experiments^36^.

### Conservation equations

The mass conservation equation for the *i*-th species is written as

4 $$\begin{aligned} \frac{dc_{i}}{dt} = -\frac{\partial J_{i}}{dx} - {\dot{r}}_{i} - J_{a,i} \end{aligned}$$

in which $c_{i}$ is the concentration in mol/m$^3$, $J_{i}$ the diffusive flux in mol/m$^2$s, ${\dot{r}}_{i}$ the consumption rate in mol/m$^3$s and $J_{a,i}$ the absorption flux into the extracellular matrix of the tissue and cells of the *i*-th compound in mol/m$^3$s. The diffusion flux depends on the chemical potential gradient $\frac{\partial \mu _{i}}{\partial x}$ in J/mol m. The diffusive flux is given by^31^

5 $$\begin{aligned} J_{i}(x,t) = - \frac{D_{i}}{RT}c_{i}\frac{\partial \mu _{i}}{\partial x} \end{aligned}$$

Here R is the gas constant in J/mol K, $D_{i}(x,t)$ [m$^2$/s] is an effective diffusion coefficient that depends on position and time due to the tortuosity of the channels. It is given by $D_{i}= D_{0,i}/[1 + (\frac{\partial h}{\partial x})^{2}]^{1/2}$^37^, with $D_{0,i}$ the diffusion coefficient of the chemical compound not affected by constrictions. The boundary conditions for the flux is:

6 $$\begin{aligned} J_{i}(0,t) = KF_{ch} \end{aligned}$$

with *K* a constant and the chemical force (transport coefficient in m/s) $F_{ch}= - K(c_{i}(0,t)-c_{i}^{*})$ with $c_{i}^{*}$ the controlled fixed value for the concentration of the *i*-th chemical compound at the boundary. The second boundary condition for the flux is $J_{i}(\infty ,t)=0$. The chemical potential of the *i*-th specie is given by

7 $$\begin{aligned} \mu _{i}(r,t) = k_{B}T \ln { c_{i}(r,t)} + k_{B}T\ln \left( \frac{ h(r,t)}{R_{m}} \right) ^{2} \end{aligned}$$

where the first term on the right-hand side corresponds to the case of the absence of constrictions, while the second term is the consequence of the effect of constrictions on transport^37^.

The absorption flux $J_{a,i}$ is proportional to the difference in the concentration of the *i*-th compound in the interstices and in the cells and extracellular matrix

8 $$\begin{aligned} J_{a,i}(r,t) = -\frac{D_{a}}{h(r,t)}(c_{i,c}-c_{i}) \end{aligned}$$

where $c_{i,c}$ is the concentration in the cells and $D_{a}$ an absorption coefficient in m/s. The consumption rate ${\dot{r}}_{i}$ is given by

9 $$\begin{aligned} {\dot{r}}_{i} = -kc_{i} \end{aligned}$$

where *k* is a kinetic constant in s$^{-1}$.

### Energy dissipation

We will compute the energy dissipated from the entropy production rate $\sigma$ [w/m$^3$k]. The entropy production rate $\sigma _{ch}$ including contributions due to diffusion, absorption and chemical reactions^38,39^ is given by

10 $$\begin{aligned} \sigma _{ch}(x,t;Y,\phi ) = -\frac{1}{T}\left[ J\frac{\partial \mu }{\partial r} + \sum _{n} J_{a,n}\Delta _{a} z + \sum _{m}J_{r,m} \Delta z_{m} \right] \end{aligned}$$

with $\Delta _{a} z_{n}$ the difference of fugacities between free and absorbed states [J/mol], $J_{r,m}$ the reaction flux of reaction *m*, and $\Delta z_{m}$ its fugacity difference.

Regarding the mechanical force, we assume that $F_{m} = \Delta P\sin {\omega t}/\lambda$, and the stretching rate is $J_{m} = -\kappa F_{m}$ [m/s]^38^, where $\kappa = \Lambda ^{-1} \sqrt{Y/\delta }/\lambda$ [m$^2$/Pa.s] is the corresponding permittivity^35^, with $\Lambda$ the attenuation coefficient [Pa/m], $\lambda$ a characteristic length [m] (cell diameter), and $\delta$ the tissue density [kg/m$^3$]. The local entropy production rate due to the mechanical force $\sigma _{m}$ is given by^38^

11 $$\begin{aligned} \sigma _{m}(x,t;Y,\phi ) = \kappa (\Delta P\sin {\omega t}/\lambda )^2 \end{aligned}$$

The local entropy production rate $\sigma$ is then $\sigma = \sigma _{ch}+\sigma _{m}$. From this quantity, we compute the total entropy produced in the tissue $\Sigma$ during the action of the external forces by integrating in space and time:

12 $$\begin{aligned} \Sigma (Y,\phi ) = \int _{0}^{\infty }\int _{0}^{\infty } \sigma (x,t;Y,\phi ) dxdt \end{aligned}$$

The energy dissipated is finally defined as $E_{d}(Y,\phi ) = T\Sigma (Y,\phi )$. Specifically, in the case of adenocarcinoma the porosity of the tissue $\phi$ can be written as a function of the Young modulus *Y* as $\phi (Y) = a(1+b\phi )^{-1}$, with *a* and *b* constants that can be obtained from experimental data^29,30^. Therefore, the dissipated energy only depends on *Y*.

### Probability of observing a given structure

According to statistical thermodynamics, the probability of observing a cancerous tissue configuration with Young’s modulus value *Y* is given by^39^

13 $$\begin{aligned} \rho (Y) \sim \exp {(\Delta G(Y)/RT)} \end{aligned}$$

where $\Delta G$ is the free energy of the tissue in J/mol, *T* its temperature and *R* the constant of gases. The ratio between probabilities of a tissue with $Y_{1}$ (e.g., healthy tissue) and another with $Y_{2}$ (e.g.,carcinogenic tissue) is thus

14 $$\begin{aligned} \frac{\rho (Y_{1})}{\rho (Y_{2})} = \exp (-\beta (\Delta G(Y_{1})-\Delta G(Y_{2}))) \end{aligned}$$

with $\beta =(RT)^{-1}$. To do this, we will consider the tissue as a self-assembled structure out of equilibrium subject to changes in free energy and energy dissipation due to the action of external forces that maintain the structure. The variation of the free energy of the tissue must incorporate a contribution due to the work that must be done on the tissue to change its structure. Thus, the free energy can be written as:

15 $$\begin{aligned} \Delta G = \Delta _{r} G + \Delta _{i} G + \Delta _{c} G \end{aligned}$$

where the first contribution, $\Delta _{r}G$, is the reversible free energy change due to the action of the force, the second term is the irreversible change (or lost work) equal to the energy dissipated per mole $E_{d}$, i.e., $\Delta _{i} G = E_{d} = T\Sigma$^40^, and the last term is the free energy cost to change the configurational parameters, i.e., Young’s modulus changes due to internal processes.

To obtain the configurational free energy change $\Delta _{c} G$, we assume that the process evolves by making efficient use of the available resources^41,42^, for which an extreme value of the energy dissipated, at which $\left. \partial \Delta E_{d}/\partial Y \right| _{Y^{*}} = 0$, is the signature of an optimal design of the structure. Therefore, the energy cost to keep a configuration different from the hypothetical efficient configuration is $\Delta _{c}G = |E_{d}(Y)-E_{d}(Y^{*})|$. By performing an expansion of $E_{d}$ around $Y^{*}$^43^, we obtain:

16 $$\begin{aligned} \Delta _{c} G \approx \frac{1}{2} \frac{\left( \partial _{Y}E_{d}\right) ^2}{| \partial _{YY}E_{d}(Y^{*})|} \end{aligned}$$

Notice that in the case of a non-dissipative tissue, i.e., $E_{d}=0$, the free energy $\Delta G$ coincides with the reversible free energy for all values of *Y*.

### Transition probability between cancer stages

We will assume that the evolution of cancer through its stages can be described by a drift-diffusion process in Y-space in which the corresponding flux is given by

17 $$\begin{aligned} J(Y) = -D_{Y}\left( \frac{\partial \rho }{\partial Y} + \frac{\rho }{RT} \frac{\partial \Delta G}{\partial Y}\right) \end{aligned}$$

with $D_{Y}$ a diffusivity in *Y*-space. Knowledge of the current is useful to characterise and delimit the different stages of cancer as a function of Young’s modulus. The boundaries between stages are characterised by an extreme value of the current derivative. A derivative equal to zero means a dynamically stable state in which the probability does not change. A negative current value indicates an unfavourable transition from one stage to the next, while high positive currents show just the opposite.

To estimate the transition probability $W_{s_i\rightarrow s_{i+1}}$ from stage $s_{i}$ to stage $s_{i+1}$, we compute the average current along both stages

18 $$\begin{aligned} \langle J \rangle _{s_{i}\rightarrow s_{i+1}} = \int _{Y_{i}}^{Y_{i+1}} J(Y)dY \end{aligned}$$

and relate it with the transition probability, observing that: i) when the average flux is equal to zero, it is equal to 0.5; ii) when the average flux is much larger than 0, it tends to 1; iii) when the average flux is much smaller than 0, it tends to 0; iv) the transition probability should depend on the average value of the derivative of the flux along the stages. The function fulfilling these requirements is a hyperbolic tangent, therefore one has

19 $$\begin{aligned} W_{s_{i}\rightarrow s_{i+1}} = \frac{1}{2}\left( 1 + \tanh (k_{i}\langle J \rangle _{s_{i}\rightarrow s_{i+1}}) \right) \end{aligned}$$

with *k* a constant that depends on the average derivative of the current at the $i^{th}$ stage. Finally, the conditional probability of being at stage *i* (for $i>1$) when starting in the healthy stage ($i=1$) is:

20 $$\begin{aligned} P_{i} = \prod _{j=2}^{j=i} W_{s_{j-1}\rightarrow s_{j}} \end{aligned}$$

where the probability of staying at the initial stage is

21 $$\begin{aligned} P_{1} = 1 - W_{s_{1}\rightarrow s_{2}} \end{aligned}$$

## Data Availability

All data generated or analysed during this study are included in this published article.
